# Targeting miR-25 to alleviate DMD-related muscle dysfunction

**DOI:** 10.1016/j.omtn.2024.102238

**Published:** 2024-06-17

**Authors:** Shuang Li, Renzhi Han

**Affiliations:** 1 Department of Pediatrics, Herman B. Wells Center for Pediatric Research, Indiana University School of Medicine, Indianapolis, IN 46202, USA

## Main text

Therapeutic approaches for Duchenne muscular dystrophy (DMD) aim to restore dystrophin expression and alleviate secondary pathology.^1^ A recent study^2^ published in Molecular Therapy-Nucleic Acids reported a promising therapeutic strategy for DMD via targeting microRNA-25 (miR-25). Kepreotis et al. demonstrated that systemic delivery of an miR-25 tough decoy (TuD) using adeno-associated virus serotype 9 (AAV9) ameliorated cardiac dysfunction in an aged mdx/utrn (+/−) mouse model. Additionally, intramuscular delivery of miR-25 TuD significantly enhanced skeletal muscle performance. This study highlights an important role of miR-25 in DMD pathology, presenting a new therapeutic option.

DMD is characterized by the absence of functional dystrophin protein due to mutations in the *DMD* gene, resulting in progressive muscle degeneration and weakness, along with severe cardiac and respiratory complications, ultimately leading to premature mortality. Encouragingly, AAV-delivered micro-dystrophin gene therapy and exon skipping therapies aiming to restore dystrophin expression have received US Food and Drug Administration (FDA) approval. However, none of these approved therapies can fully cure the disease. It is thus important to develop alternative strategies that could help to resolve some of the secondary pathologies. In this line, the FDA has recently granted approval of vamorolone, a dissociative anti-inflammatory corticosteroid, and givinostat, a histone deacetylase inhibitor, to reduce muscle inflammation and promote muscle regeneration in DMD patients.

The authors previously identified miR-25 as a suppressor of the sarcoplasmic reticulum ATPase 2a (SERCA2a).^3^ SERCA2a plays a critical role in cardiac function by actively transporting Ca^2+^ into the sarcoplasmic reticulum (SR), thereby regulating cytosolic Ca^2+^ concentration, SR Ca^2+^ load, and the rate of contraction and relaxation of the heart. The activity of SERCA2a is under a delicate regulation by a number of small-molecular-weight proteins, including phospholamban, sarcolipin, dwarf open reading frame (also known as small transmembrane regulator of ion transport 1), myoregulin, endoregulin (small integral membrane protein 6), and another-regulin. Reduced expression levels of SERCA2a have been observed in a variety of pathological conditions, including muscular dystrophy.

SERCA2a downregulation with concomitant miR-25 upregulation was observed in DMD myocytes of both dog and mouse, suggesting a potential pathogenic role of miR-25 in DMD. Systemic delivery of a low dose of AAV9 miR-25 TuD (1E11 vg per mouse) to aged mdx/utrn (+/−) mice efficiently suppressed the expression of miR-25 in cardiac tissue, leading to increased expression of SERCA2a and phosphorylation of phospholamban. This treatment also greatly improved calcium handling, cardiac function, and survival rate of mdx/utrn (+/−) mice, with reduced cardiac stress markers and apoptosis signaling.

Interestingly, cardiac fibrosis was significantly attenuated by AAV9 miR-25 TuD treatment. The authors further identified Smad7 as a direct target of miR-25, regulating transforming growth factor β (TGF-β)-mediated fibrotic signaling. Smad7 was found to be dramatically down-regulated in dystrophic dogs and mice, while AAV9 miR-25 TuD restored Smad7 expression and inhibited TGF-β signaling involved in fibrosis. A similar functional and histopathological improvement was observed in mdx/utrn (+/−) mice treated with AAV9 miR-25 TuD through intramuscular injection at 1E11 vg per mouse.

In addition to miR-25, other miRNAs have also been reported to have altered expression in DMD muscles. For example, muscle-specific miRNAs, such as miR-1, miR-133, and miR-206, are all down-regulated in DMD muscles compared to healthy controls. Interestingly, these miRNAs are highly elevated in the serum of DMD patients, likely due to increased muscle injury. In addition, several non-muscle-specific miRNAs, including miR-30c, miR-181a, and miR-95, are also found to be elevated in the serum of DMD patients.^4^ The serum elevation of these miRNAs in patients makes them potential non-protein biomarkers and therapeutic targets for DMD. Indeed, preclinical studies have demonstrated that modulating specific miRNAs can significantly impact fibrosis, inflammation, and/or muscle regeneration in DMD. For example, upregulating miR-29 and miR-146 or inhibiting miR-206 and miR-21 reduced fibrosis and inflammation. In addition, upregulating miR-29, miR-431, and miR-127 or inhibiting miR-31 has been shown to improve muscle pathology in DMD mouse models through enhancing muscle regeneration.^5^ Future research into the specific role of various miRNAs in DMD pathogenesis and their potential as therapeutic targets would provide innovative treatments for mitigating secondary pathologies in DMD.

Taken together, the study by Kepreotis et al. adds miR-25 to the growing list of miRNA-based therapeutic strategies for DMD. Although AAV9-based microdystrophin gene therapy was recently approved for DMD, the therapeutic benefits in DMD patients may not be sufficient. As evidenced in aged *mdx* mice with established dilated cardiomyopathy, AAV9-delivered microdystrophin failed to mitigate cardiac fibrosis or improve cardiac function, suggesting that microdystrophin restoration alone may be insufficient to ameliorate established DMD-related cardiac dysfunction. It would be interesting to study whether miR-25 remains elevated in dystrophic muscle and heart following AAV9-delivered microdystrophin. If so, a combination therapy with AAV9-delivered microdystrophin and miR-25 TuD may present a promising route to improve skeletal and cardiac muscle function in DMD.
